# Sex Differences in Early Functional Outcomes After Acute Stroke: A Retrospective Cohort Study at a United Kingdom Tertiary Stroke Centre

**DOI:** 10.7759/cureus.116427

**Published:** 2026-09-17

**Authors:** Saraa Zarin, Jouher Kallingal

**Affiliations:** 1 Neurology, Salford Royal National Health Service (NHS) Foundation Trust, Salford, GBR; 2 Faculty of Biology, Medicine and Health, University of Manchester, Manchester, GBR; 3 Stroke Medicine, Salford Royal National Health Service (NHS) Foundation Trust, Salford, GBR

**Keywords:** functional outcome, modified rankin scale, premorbid disability, retrospective cohort, sex differences, stroke, stroke severity

## Abstract

Background

Women are frequently reported to have poorer functional outcomes after stroke than men, but women with stroke are also often older and may have greater functional impairment before the index event. We examined sex differences in early functional outcomes after acute stroke and assessed whether any observed association persisted after accounting for age, pre-stroke disability, stroke severity, stroke subtype, and relevant comorbidities.

Materials and methods

We conducted a retrospective cohort study of 1,252 consecutive adults admitted with acute ischaemic stroke or primary intracerebral haemorrhage to a tertiary stroke centre in the United Kingdom between June and December 2023. The primary outcome was functional status at hospital discharge across the full modified Rankin Scale (mRS; 0-6). Cumulative-link ordinal logistic regression was used to examine the association between female sex and worse discharge mRS. Sequential models adjusted for age, pre-stroke mRS, admission National Institutes of Health Stroke Scale (NIHSS) score, stroke subtype, and prespecified comorbidities. Sensitivity analyses included patients with ischaemic stroke only, patients with pre-stroke mRS 0-2, dichotomised functional outcomes, and in-hospital mortality.

Results

The cohort comprised 601 women (48.0%) and 651 men (52.0%). Women were older than men (median 77 years (interquartile range (IQR) 66-85) vs 72 years (IQR 62-81), p<0.001) and had greater pre-stroke functional impairment (median pre-stroke mRS 2 (IQR 1-3) vs 1 (IQR 0-2), p<0.001). Discharge mRS of 3-6 occurred in 405 women (67.4%) and 391 men (60.1%; unadjusted odds ratio (OR) 1.37, 95% confidence interval (CI) 1.09-1.73; p=0.008). In the primary ordinal analysis, female sex was associated with worse discharge mRS before adjustment (common OR 1.34, 95% CI 1.10-1.64; p=0.003). The association attenuated after adjustment for age (OR 1.13, 95% CI 0.92-1.37; p=0.247) and moved close to the null after additional adjustment for pre-stroke mRS (OR 0.95, 95% CI 0.77-1.16; p=0.604). The fully adjusted common OR was 0.94 (95% CI 0.76-1.16; p=0.568). Sensitivity analyses produced similar findings.

Conclusions

Women had poorer unadjusted functional status at discharge after acute stroke, but this association was substantially attenuated after accounting for age and pre-stroke functional status. Female sex was not independently associated with worse early functional outcome after multivariable adjustment. These findings emphasise the importance of premorbid disability and baseline case mix when interpreting apparent sex differences in early stroke outcomes.

## Introduction

Stroke remains a major cause of death and disability worldwide, with a substantial and increasing absolute burden as populations grow and age [1]. Differences between women and men have been described across stroke epidemiology, presentation, treatment, recovery, and functional outcome. Women often experience poorer unadjusted post-stroke outcomes, but they also tend to be older at stroke onset and may have greater pre-existing disability and a different clinical risk profile [2-4].

These baseline differences complicate the interpretation of post-stroke disability. In particular, premorbid functional status can confound apparent sex differences because the modified Rankin Scale (mRS) at follow-up reflects a patient's overall level of disability rather than disability attributable solely to the index stroke. Renoux and colleagues showed that adjustment for pre-stroke functional status can materially alter estimates of sex differences in stroke severity and outcome [5].

The mRS is widely used to assess global disability after stroke [6]. Although the mRS is often dichotomised into favourable and unfavourable outcomes, analysing all the ordered categories of the mRS (0-6) retains information across the range of functional outcomes that may be lost when the scale is reduced to two groups using a single threshold [7]. This is particularly relevant when comparing groups whose premorbid functional status differs.

We therefore examined sex differences in baseline characteristics and early functional outcomes in a contemporary cohort of consecutive patients admitted with acute stroke to a United Kingdom tertiary stroke centre. We hypothesised that women would have poorer unadjusted functional status at discharge, but that the association would attenuate after accounting for age, pre-stroke disability, stroke severity, stroke subtype, and relevant comorbidities. As the outcome was assessed at hospital discharge, this study specifically addresses early functional status and does not evaluate longer-term post-stroke recovery.

## Materials and methods

Study design and setting

This retrospective observational cohort study included consecutive adults admitted to the Hyper Acute Stroke Unit at Salford Royal Hospital, Greater Manchester, United Kingdom, between June 1 and December 31, 2023.

Salford Royal Hospital provides specialist hyperacute stroke care, including emergency clinical assessment, neuroimaging, intravenous thrombolysis, mechanical thrombectomy where clinically indicated, inpatient stroke-unit care, rehabilitation assessment, and discharge planning.

The study was reported in accordance with the Strengthening the Reporting of Observational Studies in Epidemiology (STROBE) recommendations [8].

Participants

Patients were eligible if they were aged 18 years or older, had a confirmed diagnosis of acute ischaemic stroke or primary intracerebral haemorrhage, and had functional status recorded at discharge.

Patients with transient ischaemic attack without confirmed stroke, stroke mimics, traumatic intracranial haemorrhage, subarachnoid haemorrhage, or cerebral venous thrombosis were not included. For patients with more than one eligible stroke admission during the study period, only the first admission was included so that each patient contributed one observation.

A total of 1,252 patients met the eligibility criteria and were included in the study.

Data source and variables

Anonymised clinical information was obtained retrospectively from routinely collected electronic health records. Variables available for analysis included age, recorded sex, congestive heart failure, hypertension, atrial fibrillation, diabetes mellitus, previous transient ischaemic attack, dementia, pre-stroke mRS, National Institutes of Health Stroke Scale (NIHSS) score on arrival [9,10], stroke subtype, and discharge mRS.

Stroke subtype was classified as acute ischaemic stroke or primary intracerebral haemorrhage. One patient had an unclassified stroke subtype. The exposure of interest was recorded sex, analysed as female versus male, with male sex used as the reference group.

Outcomes

The primary outcome was functional status at hospital discharge assessed using the full modified Rankin Scale (mRS), ranging from 0 (no symptoms) to 6 (death) [11]. Discharge mRS was routinely recorded in the electronic patient record as a mandatory component of the discharge process. Higher scores indicate increasing disability, with mRS 6 representing death. The primary analysis, therefore, examined whether female sex was associated with higher odds of being in a worse discharge mRS category.

Secondary outcomes included dichotomised functional status at hospital discharge (mRS of 3-6 versus 0-2 and mRS of 4-6 versus 0-3), in-hospital mortality, and new dependency or death among patients with a pre-stroke mRS of 0-2. New dependency or death was defined as discharge mRS of 3-6 among patients who had a pre-stroke mRS of 0-2. The pre-stroke mRS of 0-2 subgroup was examined to reduce the influence of substantial pre-existing dependency on the discharge outcome.

Statistical analysis

Continuous variables were summarised using medians and interquartile ranges, and categorical variables using counts and percentages. Baseline characteristics were compared between women and men using the Wilcoxon rank-sum test for continuous variables and Pearson's chi-squared test or Fisher's exact test for categorical variables, as appropriate. Absolute standardised mean differences (SMDs) were also calculated to describe the magnitude of between-group differences.

The primary analysis used cumulative-link ordinal logistic regression to model discharge mRS across the complete 0-6 scale. The results are reported as common ORs with 95% CIs. An OR greater than 1 indicates higher odds of being in a worse discharge mRS category among women compared with men.

Five sequential models were fitted in the common analytic cohort of 1,251 patients with classified stroke subtype. Model 1 included sex alone. Model 2 additionally adjusted for age. Model 3 additionally included pre-stroke mRS. Model 4 further adjusted for admission NIHSS and stroke subtype. Model 5 additionally included congestive heart failure, hypertension, atrial fibrillation, diabetes mellitus, previous transient ischaemic attack, and dementia.

Age and admission NIHSS were modelled flexibly using natural cubic spline terms with four degrees of freedom rather than assuming a strictly linear relationship with discharge outcome.

Sensitivity analyses repeated the fully adjusted ordinal model among patients with ischaemic stroke only and among patients with pre-stroke mRS of 0-2. Fully adjusted binary logistic regression models were also used to examine discharge mRS of 3-6, discharge mRS of 4-6, in-hospital mortality, and new dependency or death among patients with pre-stroke mRS of 0-2.

The proportional-odds assumption for the sex effect was assessed graphically. Missing data were minimal. Of the 1,252 included patients, one had an unclassified stroke subtype and was excluded from models requiring stroke subtype, resulting in an analytic cohort of 1,251 patients. No imputation was performed.

All analyses were performed using R version 4.5.3 (R Foundation for Statistical Computing, Vienna, Austria). Statistical tests were two-sided and a p-value <0.05 was considered statistically significant.

## Results

Study population and baseline characteristics

The study included 1,252 patients, of whom 601 (48.0%) were women and 651 (52.0%) were men. Women were older than men, with a median age of 77 years (IQR 66-85) compared with 72 years (IQR 62-81; p<0.001; absolute SMD 0.24). Women also had greater premorbid functional impairment, with a median pre-stroke mRS of 2 (IQR 1-3), compared with 1 (IQR 0-2) among men (p<0.001; absolute SMD 0.34).

Median NIHSS on arrival was 6 (IQR 3-14) in women and 6 (IQR 3-12) in men (p=0.057). Other measured vascular risk factors and comorbidities were broadly similar between the groups. Primary intracerebral haemorrhage occurred in 66 women (11.0%) and 93 men (14.3%; p=0.081). Baseline characteristics are shown in Table 1.

**Table 1:** Baseline Clinical Characteristics According to Sex Data are median (IQR) or n (%) unless otherwise stated. SMD, standardised mean difference; NIHSS, National Institutes of Health Stroke Scale.

Characteristic	Women	Men	Absolute SMD	P value
Age, years	77 (66–85)	72 (62–81)	0.24	<0.001
Pre-stroke modified Rankin Scale	2 (1–3)	1 (0–2)	0.34	<0.001
NIHSS on arrival	6 (3–14)	6 (3–12)	0.13	0.057
Congestive heart failure	35 (5.8%)	27 (4.1%)	0.08	0.172
Hypertension	324 (53.9%)	347 (53.3%)	0.01	0.829
Atrial fibrillation	119 (19.8%)	119 (18.3%)	0.04	0.493
Diabetes mellitus	139 (23.1%)	174 (26.7%)	0.08	0.142
Previous transient ischaemic attack	126 (21.0%)	139 (21.4%)	0.01	0.867
Dementia	50 (8.3%)	40 (6.1%)	0.08	0.137
Primary intracerebral haemorrhage	66 (11.0%)	93 (14.3%)	0.1	0.081

Early functional outcomes

Median discharge mRS was 3 (IQR 2-4) in both women and men, although the overall distribution across the mRS differed between groups (p=0.003). Discharge mRS of 3-6 occurred in 405 women (67.4%) and 391 men (60.1%), giving an unadjusted OR of 1.37 (95% CI 1.09-1.73; p=0.008). Discharge mRS of 4-6 occurred in 287 women (47.8%) and 267 men (41.0%; OR 1.31, 95% CI 1.05-1.64; p=0.017).

In-hospital death occurred in 60 women (10.0%) and 50 men (7.7%). The difference was not statistically significant (OR 1.33, 95% CI 0.90-1.97; p=0.162). Among patients with pre-stroke mRS 0-2, new dependency or death occurred in 194 women (50.5%) and 238 men (48.3%; OR 1.09, 95% CI 0.84-1.43; p=0.540). Early functional outcomes are summarised in Table 2.

**Table 2:** Early Functional Outcomes According to Sex Data are N (%) unless otherwise stated. Women: N=601; men: N=651. For the pre-stroke mRS 0-2 subgroup, women: N=384; men: N=493. ORs compare women with men. The p value for median discharge mRS relates to the overall distributional comparison; binary outcome ORs are unadjusted. mRS, modified Rankin Scale; IQR, interquartile range; OR, odds ratio; CI, confidence interval.

Outcome	Women	Men	Unadjusted OR (95% CI)	P value
Discharge mRS, median (IQR)	3 (2-4)	3 (2-4)	-	0.003
Discharge mRS of 3-6	405 (67.4%)	391 (60.1%)	1.37 (1.09–1.73)	0.008
Discharge mRS of 4-6	287 (47.8%)	267 (41.0%)	1.31 (1.05–1.64)	0.017
In-hospital death	60 (10.0%)	50 (7.7%)	1.33 (0.90–1.97)	0.162
New dependency or death among patients with pre-stroke mRS of 0-2	194 (50.5%)	238 (48.3%)	1.09 (0.84–1.43)	0.54

**Figure 1:**
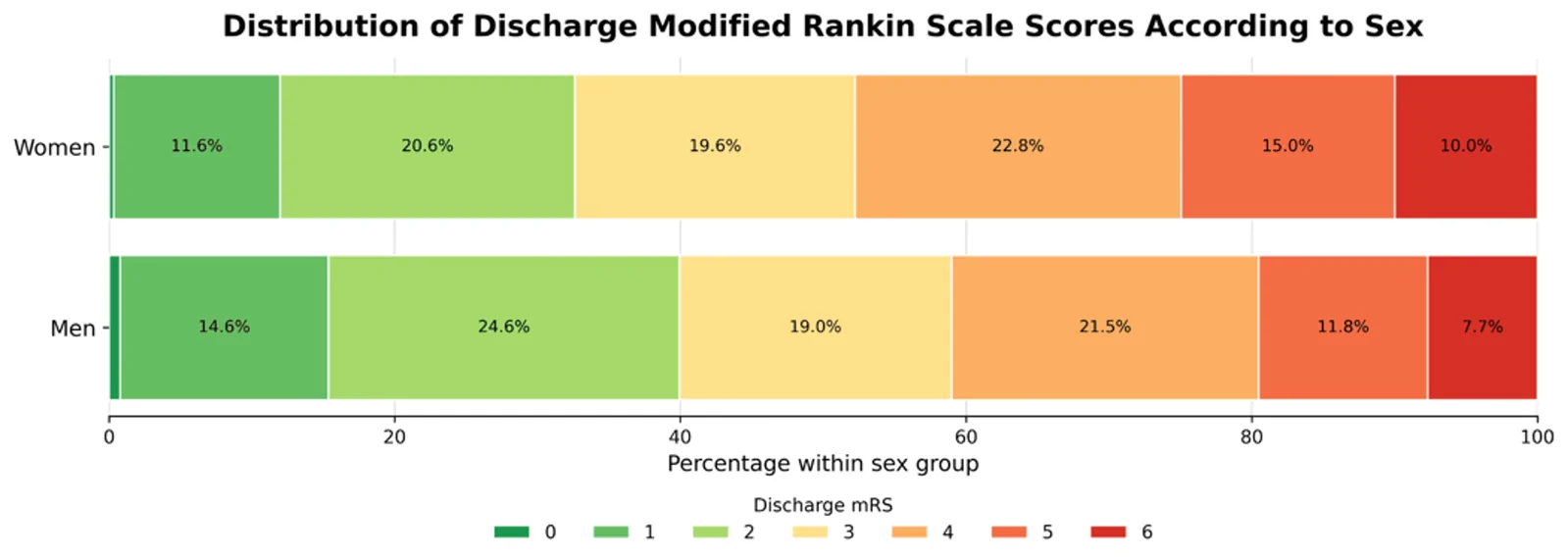
Distribution of Discharge Modified Rankin Scale Scores According to Sex. Stacked bars show the percentage distribution of discharge mRS scores from 0 to 6 among women and men. Higher mRS scores indicate greater functional impairment, with mRS 6 representing death.

The complete distribution of discharge mRS scores is shown in Figure 1.

Women had a greater proportion of patients in higher disability categories, particularly mRS of 5 and 6, whereas men had a greater proportion in lower mRS categories.

Sequential ordinal regression analysis

In the unadjusted ordinal model, female sex was associated with higher odds of being in a worse discharge mRS category (common OR 1.34, 95% CI 1.10-1.64; p=0.003). Adjustment for age substantially attenuated this association (common OR 1.13, 95% CI 0.92-1.37; p=0.247). After further adjustment for pre-stroke mRS, the estimate moved close to the null (common OR 0.95, 95% CI 0.77-1.16; p=0.604).

Additional adjustment for admission NIHSS and stroke subtype resulted in little further change (common OR 0.94, 95% CI 0.77-1.16; p=0.593), and the fully adjusted estimate remained similar (common OR 0.94, 95% CI 0.76-1.16; p=0.568). The greatest attenuation in the association between female sex and discharge mRS therefore occurred after accounting for age and pre-stroke functional status, while subsequent adjustment for stroke severity, stroke subtype, and measured comorbidities had comparatively little additional effect. The sequential ordinal regression models are summarised in Table 3.

**Table 3:** Sequential Ordinal Regression Models for the Association Between Female Sex and Discharge mRS A common OR greater than 1 indicates higher odds of being in a worse discharge mRS category among women. The fully adjusted model included age, pre-stroke mRS, admission NIHSS, stroke subtype, congestive heart failure, hypertension, atrial fibrillation, diabetes mellitus, previous transient ischaemic attack, and dementia. mRS, modified Rankin Scale; OR, odds ratio; CI, confidence interval; NIHSS, National Institutes of Health Stroke Scale.

Model	N	Common OR	95% CI	P value
Unadjusted	1251	1.34	1.10-1.64	0.003
+ Age	1251	1.13	0.92-1.37	0.247
+ Age + pre-stroke mRS	1251	0.95	0.77-1.16	0.604
+ Age + pre-stroke mRS + NIHSS + stroke subtype	1251	0.94	0.77-1.16	0.593
Fully adjusted	1251	0.94	0.76-1.16	0.568

**Figure 2:**
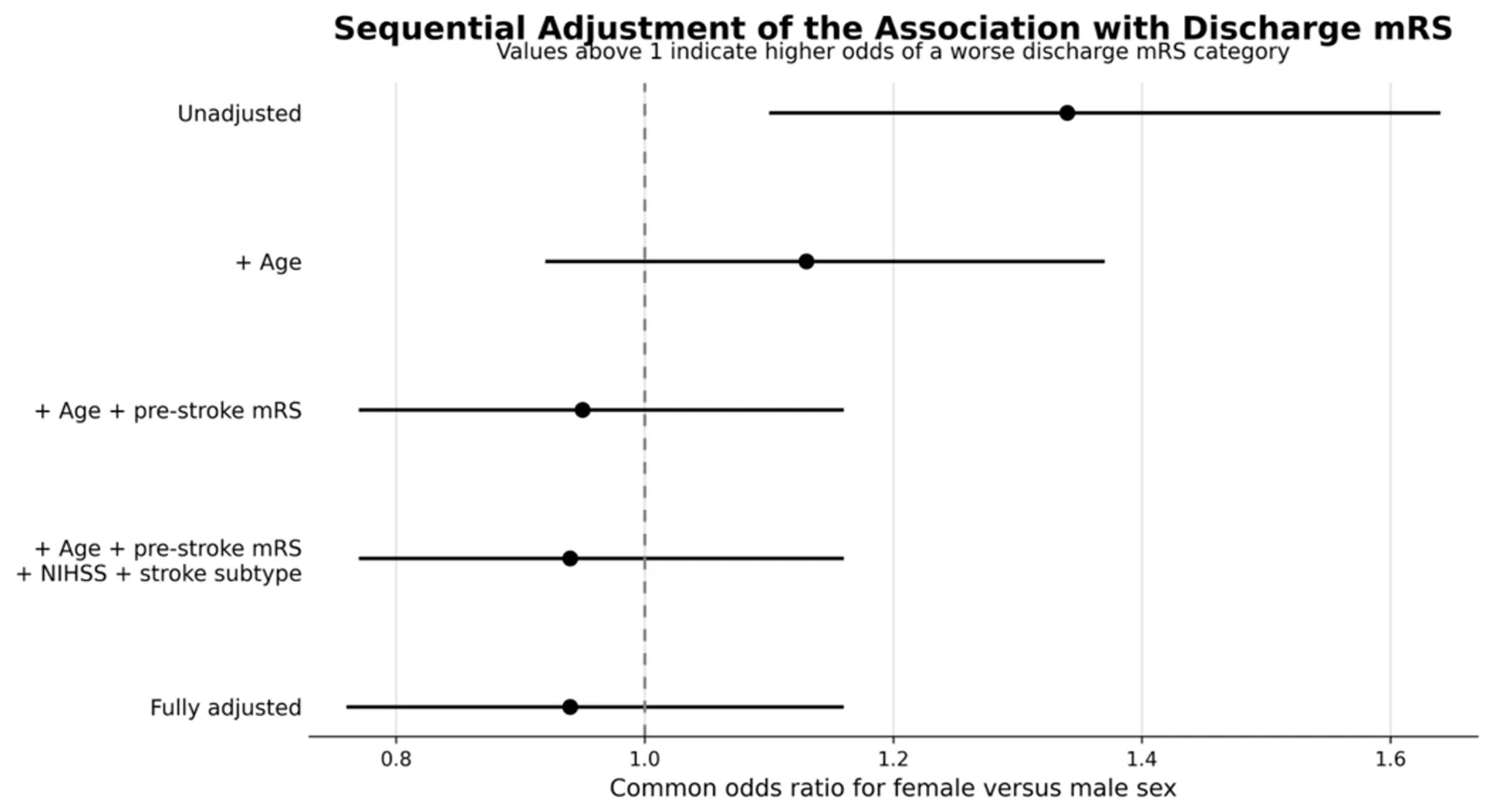
Sequential Adjustment of the Association With Discharge Modified Rankin Scale. Points represent common odds ratios for female versus male sex and horizontal lines represent 95% confidence intervals. Values above 1 indicate higher odds of being in a worse discharge mRS category among women. The dashed vertical line indicates the null value of 1.

The sequential change in the association across adjustment models is illustrated in Figure 2.

Fully adjusted and sensitivity analyses

Within the fully adjusted model, pre-stroke mRS remained strongly associated with worse discharge functional status (OR per one-point increase 1.89, 95% CI 1.73-2.07; p<0.001). Age and admission NIHSS were also associated with discharge mRS when modelled flexibly (both p<0.001). Primary intracerebral haemorrhage was associated with worse discharge mRS compared with ischaemic stroke (OR 2.17, 95% CI 1.58-2.99; p<0.001). Full model estimates are provided in Table 4.

**Table 4:** Fully Adjusted Ordinal Regression Model for Discharge Modified Rankin Scale Age and admission NIHSS were modelled using natural cubic splines; a single odds ratio is therefore not reported for these terms. ORs greater than 1 indicate higher odds of a worse discharge mRS category. mRS=modified Rankin Scale; NIHSS=National Institutes of Health Stroke Scale; OR=odds ratio; CI=confidence interval; df=degrees of freedom.

Variable	Modelling note	OR	95% CI	P value
Female sex	-	0.94	0.76-1.16	0.568
Age	Natural cubic spline (4 df)	-	-	<0.001
Pre-stroke mRS, per point	-	1.89	1.73-2.07	<0.001
Admission NIHSS	Natural cubic spline (4 df)	—	—	<0.001
Primary intracerebral haemorrhage vs ischaemic stroke	-	2.17	1.58-2.99	<0.001
Congestive heart failure	-	0.85	0.53-1.37	0.507
Hypertension	-	0.96	0.78-1.19	0.734
Atrial fibrillation	-	1.02	0.78-1.34	0.874
Diabetes mellitus	-	1.16	0.92-1.47	0.214
Previous transient ischaemic attack	-	0.82	0.63-1.06	0.126
Dementia	-	0.98	0.65-1.47	0.913

Sensitivity analyses were consistent with the primary result. Among patients with ischaemic stroke, the adjusted common OR for female versus male sex was 0.90 (95% CI 0.72-1.12; p=0.354). Among patients with pre-stroke mRS of 0-2, the adjusted common OR was 0.94 (95% CI 0.73-1.21; p=0.628). Female sex was also not independently associated with discharge mRS of 3-6 (adjusted OR 0.87, 95% CI 0.62-1.21; p=0.403), discharge mRS of 4-6 (OR 0.89, 95% CI 0.66-1.20; p=0.454), in-hospital mortality (OR 0.90, 95% CI 0.57-1.43; p=0.661), or new dependency or death among patients with pre-stroke mRS of 0-2 (OR 0.90, 95% CI 0.64-1.27; p=0.551). These analyses are summarised in Table 5.

**Table 5:** Sensitivity Analyses of the Association Between Female Sex and Early Functional Outcomes Percentages are calculated using the primary analytic cohort of 1,251 patients as the denominator. For ordinal models, estimates are adjusted common odds ratios; for binary logistic regression models, estimates are adjusted odds ratios. All estimates compare women with men. mRS=modified Rankin Scale; OR=odds ratio; CI=confidence interval.

Analysis	N (%)	Outcome/model	Adjusted OR (95% CI)	P value
Primary analytic cohort	1251 (100%)	Ordinal discharge mRS 0-6	0.94 (0.76–1.16)	0.568
Ischaemic stroke only	1092 (87.3%)	Ordinal discharge mRS 0-6	0.90 (0.72–1.12)	0.354
Pre-stroke mRS 0-2	877 (70.1%)	Ordinal discharge mRS 0-6	0.94 (0.73–1.21)	0.628
Discharge mRS 3-6	1251 (100%)	Binary logistic regression	0.87 (0.62–1.21)	0.403
Discharge mRS 4-6	1251 (100%)	Binary logistic regression	0.89 (0.66–1.20)	0.454
In-hospital death	1251 (100%)	Binary logistic regression	0.90 (0.57–1.43)	0.661
New dependency/death, pre-stroke mRS 0-2	877 (70.1%)	Binary logistic regression	0.90 (0.64–1.27)	0.551

**Figure 3:**
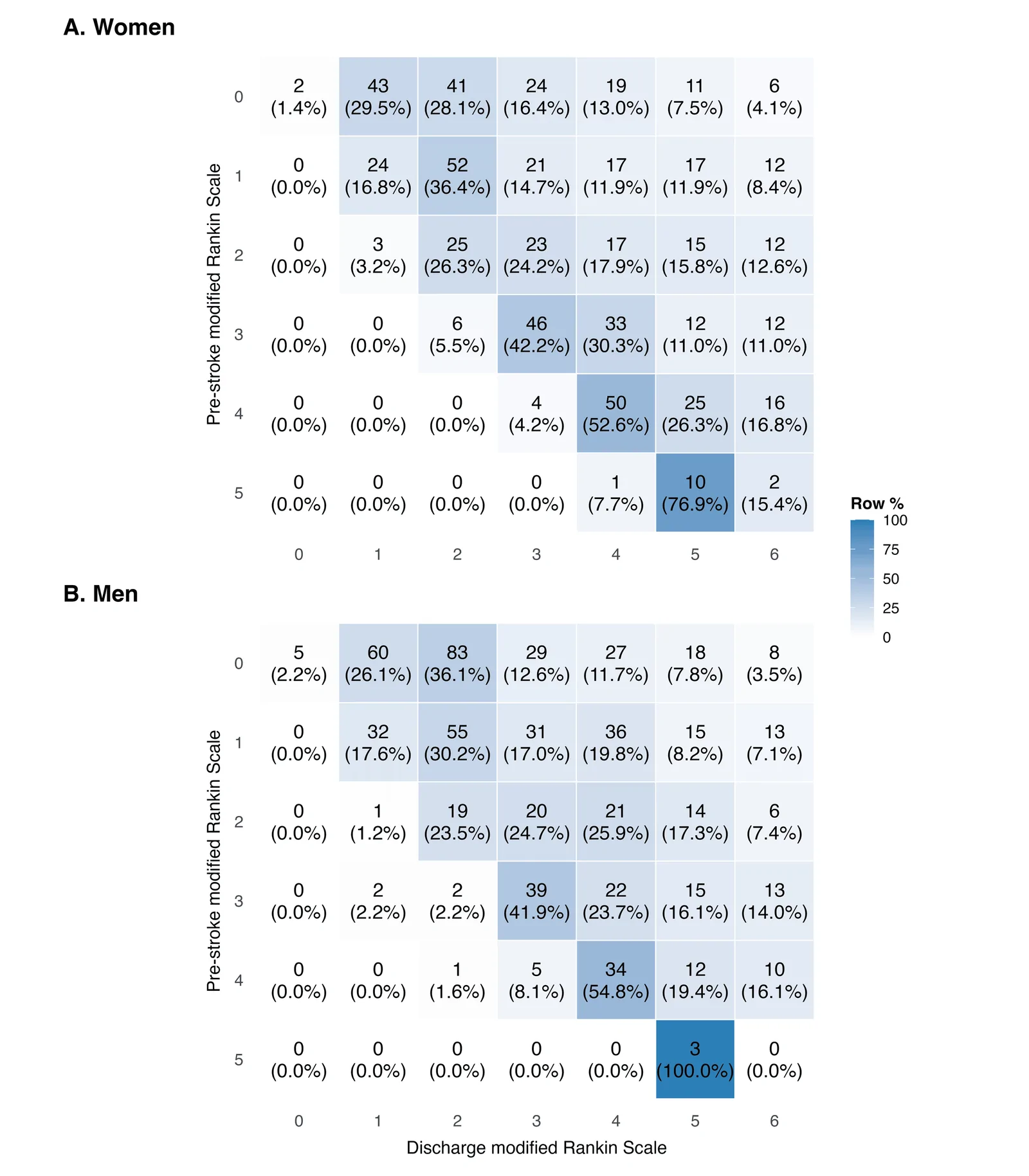
Pre-stroke-to-Discharge Modified Rankin Scale Transition According to Sex. Heatmaps show discharge mRS within each pre-stroke mRS category for women (A) and men (B). Cells display patient counts and row percentages.

Pre-stroke-to-discharge mRS transitions in women and men are displayed in Figure 3.

The transition plots illustrate that absolute functional status at discharge reflects both the index stroke and the patient's level of function before the event. The underlying transition counts are provided in Table 6.

**Table 6:** Pre-stroke-to-Discharge Modified Rankin Scale Transition Counts According to Sex Cells show patient counts for each transition from pre-stroke mRS (rows) to discharge mRS (columns). mRS=modified Rankin Scale.

Sex	Pre-stroke mRS	Discharge mRS 0	Discharge mRS 1	Discharge mRS 2	Discharge mRS 3	Discharge mRS 4	Discharge mRS 5	Discharge mRS 6
Women	0	2	43	41	24	19	11	6
Women	1	0	24	52	21	17	17	12
Women	2	0	3	25	23	17	15	12
Women	3	0	0	6	46	33	12	12
Women	4	0	0	0	4	50	25	16
Women	5	0	0	0	0	1	10	2
Men	0	5	60	83	29	27	18	8
Men	1	0	32	55	31	36	15	13
Men	2	0	1	19	20	21	14	6
Men	3	0	2	2	39	22	15	13
Men	4	0	0	1	5	34	12	10
Men	5	0	0	0	0	0	3	0

## Discussion

Principal findings

In this cohort of 1,252 consecutive acute stroke admissions, women had poorer functional status at discharge in unadjusted analyses, but female sex was not independently associated with a worse position on the mRS after adjustment. The attenuation was concentrated in the first two adjustment steps: the common OR fell from 1.34 in the unadjusted model to 1.13 after accounting for age and to 0.95 after pre-stroke mRS was added. Subsequent adjustment for admission NIHSS, stroke subtype, and measured comorbidities changed the estimate very little.

The same pattern was seen across sensitivity analyses, including ischaemic stroke only, patients with pre-stroke mRS of 0-2, dichotomised disability outcomes, and in-hospital mortality. Taken together, the findings suggest that the crude difference in early functional status between women and men in this cohort was largely explained by baseline case mix, particularly older age and poorer function before the stroke, rather than by an independent adverse association with female sex.

Comparison with previous studies

Recent studies have not shown a single consistent independent effect of sex on post-stroke functional outcome. The most relevant United Kingdom comparator is a recent national analysis from the Sentinel Stroke National Audit Programme (SSNAP). Women were older and more often had severe stroke, and after multivariable adjustment, they remained slightly more likely to have a poorer functional outcome at discharge (adjusted relative risk 1.06, 95% CI 1.05-1.06) [3]. This differs from our cohort, where the association moved close to the null after accounting for age and pre-stroke mRS. The contrast may reflect differences in population, outcome definition, care processes, and covariate structure between a national audit and a single-centre cohort.

Other studies illustrate similar heterogeneity. Renoux and colleagues found no evidence of worse outcome in women after adjustment for age and premorbid mRS [5]. A large German registry study likewise demonstrated substantial attenuation of the apparent sex difference after adjustment, with fully adjusted analyses showing slightly more favourable discharge outcomes and lower in-hospital mortality among women [12]. The Third China National Stroke Registry found worse adjusted functional outcome in women at three months but not at six or 12 months [13], while a later national registry analysis showed that the 12-month sex difference had attenuated by 2015-2018 [14]. A prospective Vietnamese multicentre registry similarly found no sex difference in 90-day functional outcome [15].

Some longer-term population studies, however, have found persistent functional differences. In the South London Stroke Register, women had better adjusted survival but poorer activities of daily living, anxiety, and health-related quality of life among survivors [4]. A recent population-based United States study also reported worse adjusted activities of daily living and instrumental activities of daily living outcomes in women during the first year after stroke, while neurologic, cognitive, and quality-of-life outcomes showed no significant sex differences [16]. Taken together, these studies suggest that the apparent effect of sex varies according to the population, outcome measure, timing of assessment, and the extent to which premorbid function and other case-mix factors are considered.

Importance of pre-stroke functional status

The sequential modelling in this study illustrates why pre-stroke function deserves particular attention. The mRS is a measure of functional state at a given time rather than a direct measure of disability newly caused by the index stroke. A patient who was already dependent before stroke may therefore have a high discharge mRS even when the additional functional loss from the acute event is modest. When pre-stroke disability differs systematically between comparison groups, an analysis of absolute post-stroke mRS can partly reflect those pre-existing differences [5].

This issue was especially relevant in our cohort because women had a median pre-stroke mRS of one point higher than in men. The sex estimate moved from 1.13 after adjustment for age to 0.95 after adding pre-stroke mRS, with little subsequent change. The analysis restricted to patients with a pre-stroke mRS of 0-2 reached the same overall conclusion. These observations do not prove that premorbid disability is the only explanation for crude sex differences, but they show that omitting it would have produced a materially different interpretation of the same cohort.

Clinical interpretation

These findings do not imply that sex-related differences are unimportant in stroke. Sex-related biology, vascular risk profiles, treatment delivery, rehabilitation, and social circumstances may all influence recovery [2,3]. Our dataset was not designed to identify those mechanisms, and the present findings should therefore be interpreted specifically as an analysis of early functional outcome after case-mix adjustment.

The more specific implication is methodological: crude comparisons of discharge disability between women and men can be misleading when age and premorbid function differ. Studies and quality-improvement analyses that compare post-stroke functional outcomes should account for pre-stroke functional status alongside age and acute stroke severity whenever those data are available. The full ordinal mRS is also useful in this setting because it preserves outcome information across the disability spectrum rather than depending on a single dichotomous cut-point [6,7].

Strengths and limitations

The principal strengths of this study are the relatively large consecutive cohort, use of the complete ordinal mRS as the primary outcome, transparent sequential adjustment, flexible modelling of age and NIHSS, and the consistency of the findings across several sensitivity analyses.

The findings should be interpreted in light of the retrospective single-centre design. Discharge mRS represents an early outcome rather than a standardised 90-day endpoint, and detailed treatment, rehabilitation, care process, and social variables were not available for modelling; therefore, residual confounding and unmeasured differences in care delivery cannot be completely excluded. In addition, recorded sex was available only as a binary variable (woman or man), which limited our ability to examine more nuanced sex- and gender-related differences. Against this, the cohort was consecutive and relatively large, the primary analysis retained the full ordinal mRS, and the same overall pattern was observed across multiple sensitivity analyses.

## Conclusions

Women in this acute stroke cohort had poorer functional status at hospital discharge in unadjusted analyses, but they were also older and had greater functional impairment before the index stroke. Adjustment for age substantially attenuated the association, and the addition of pre-stroke mRS moved the estimate close to the null. Further adjustment for stroke severity, stroke subtype, and measured comorbidities produced little additional change.

Female sex was therefore not independently associated with worse early functional status in this cohort, a finding that was consistent across sensitivity analyses. The results emphasise that apparent sex differences in post-stroke disability should be interpreted in the context of premorbid function and baseline case mix rather than from crude discharge outcomes alone.
